# The Crystal Structure of Pneumolysin at 2.0 Å Resolution Reveals the Molecular Packing of the Pre-pore Complex

**DOI:** 10.1038/srep13293

**Published:** 2015-09-03

**Authors:** Jamie E. Marshall, Bayan H. A. Faraj, Alexandre R. Gingras, Rana Lonnen, Md. Arif Sheikh, Mohammed El-Mezgueldi, Peter C. E. Moody, Peter W. Andrew, Russell Wallis

**Affiliations:** 1 Department of Infection, Immunity and Inflammation, University of Leicester, PO Box 138, Leicester, LE1 9HN UK; 2 Department of Biochemistry, University of Leicester, PO Box 138, Leicester, LE1 9HN UK

## Abstract

Pneumolysin is a cholesterol-dependent cytolysin (CDC) and virulence factor of *Streptococcus pneumoniae*. It kills cells by forming pores assembled from oligomeric rings in cholesterol-containing membranes. Cryo-EM has revealed the structures of the membrane-surface bound pre-pore and inserted-pore oligomers, however the molecular contacts that mediate these oligomers are unknown because high-resolution information is not available. Here we have determined the crystal structure of full-length pneumolysin at 1.98 Å resolution. In the structure, crystal contacts demonstrate the likely interactions that enable polymerisation on the cell membrane and the molecular packing of the pre-pore complex. The hemolytic activity is abrogated in mutants that disrupt these intermolecular contacts, highlighting their importance during pore formation. An additional crystal structure of the membrane-binding domain alone suggests that changes in the conformation of a tryptophan rich-loop at the base of the toxin promote monomer-monomer interactions upon membrane binding by creating new contacts. Notably, residues at the interface are conserved in other members of the CDC family, suggesting a common mechanism for pore and pre-pore assembly.

The cholesterol-dependent cytolysin (CDC), pneumolysin^1^ is a virulence factor of the human pathogen *Streptococcus pneumoniae* (pneumococcus)^2^, the leading cause of bacterial pneumonia and a major agent of meningitis and septicaemia as well as debilitating diseases such as otitis media, septic arthritis, keratitis and sinusitis^3^. It is found in virtually all pneumococcal isolates and plays key roles in bacterial colonisation, invasion and inflammation^4^,^5^,^6^. A central feature of the toxin is the formation of transmembrane pores in cholesterol-containing membranes^7^, which lyse or disrupt cell functioning, depending on the concentration^8^,^9^,^10^. Pneumolysin and other CDCs bind to mammalian membranes through a tryptophan-rich loop and a threonine-leucine pair within a membrane-binding domain at the base of the toxin^11^,^12^. The tryptophan-rich loop has been proposed to spring out of the body of the toxin on association with the membrane^12^. All three tryptophans are implicated in membrane binding but Trp433 and Trp435 are particularly important^13^,^14^. Once bound to the membrane, monomers then oligomerise to form a pre-pore complex that subsequently undergoes a complex set of structural transitions to form a transmembrane pore, typically comprising 30–50 subunits. Structures of the pre-pore and inserted-pore oligomers have been determined by cryo-EM at ~28 Å, and these structures together with recent analysis of the related CDC, suilysin, show that that pore formation follows the vertical collapse of the pre-pore structure with two extended beta-hairpins penetrating the membrane to form the large beta-barrel pore^7^,^15^.

Although high-resolution X-ray structures have been determined for several CDCs, pneumolysin has proved to be elusive. Consequently, structure-function analysis has relied on models based on the structures of the other CDC toxins. Critically, the lack of high-resolution data means that the interactions enabling pneumolysin monomers to pack together to form the pore and pre-pore complexes is not known. In this work, we have determined the structure of pneumolysin at 1.98 Å resolution. The side-by-side packing arrangement in the crystal demonstrates the packing of monomers in the pre-pore complex. Additional structural and mutagenesis data has identified residues that are likely to contribute towards oligomerisation during pre-pore formation.

## Results

### The structure of pneumolysin

**Figure 1:**
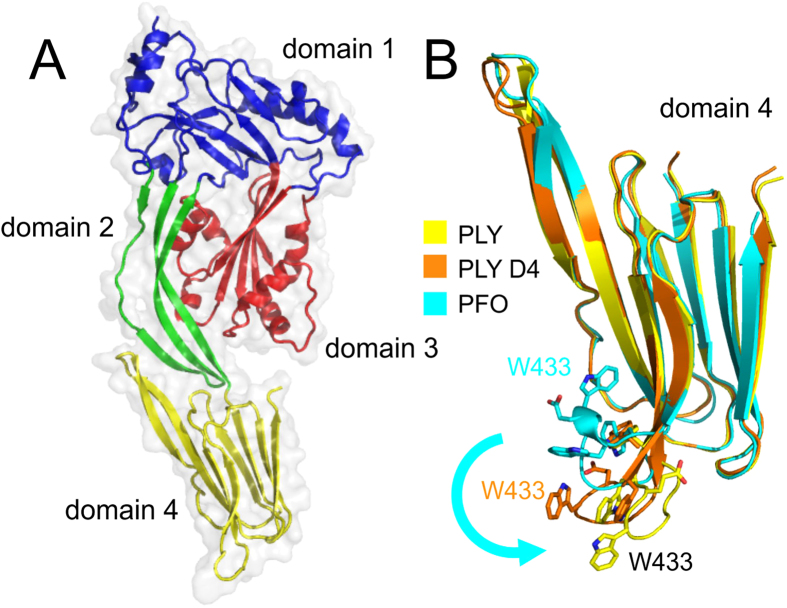
Domain structure of pneumolysin and the likely changes upon membrane binding. (**A**) Domains 1, 2 and 3 are contiguous and connected to the C-terminal membrane-binding domain (domain 4) via domain 2. (**B**) overlay of the membrane-binding domains of the full-length pneumolysin (PLY), pneumolysin domain 4 (PLY D4) and perfringolysin O (PFO; PDB: 1PFO^12^;) structures. The structures reveal the likely changes that occur upon membrane binding. Notably, the tryptophan-rich loop flips outwards and downwards (*arrow*) exposing Trp433, to enable binding to the membrane.

Full-length pneumolysin and a truncated form comprising the membrane-binding domain (domain 4) were produced by expression in *E. coli* and purified by affinity and gel filtration chromatography. Crystals of the full-length toxin were grown at 4 °C and at pH 8.5. The best crystals were obtained from protein containing substitutions Asp385Asn and Cys432Ala. Neither mutation prevents pore formation^16^,^17^. The asymmetric unit contained a single molecule having the characteristic four domain structure of a CDC, with three non-contiguous domains (domains 1–3) linked to the C-terminal membrane-binding domain (Fig. 1A) via the strap-like domain 2. Crystal of the membrane-binding domain were grown at room temperature and contained two molecules in the asymmetric unit. Both refined models have good stereochemistry (Table 1) with 95% and 93% of residues within the Ramachandran favoured regions.

The tryptophan-rich loop, projects from the base of domain 4 in the structure of full-length pneumolysin, reflecting the likely membrane-binding conformation (Fig. 1B). Notably, two of the tryptophan residues, Trp433 and Trp435, shown previously to be important for membrane binding and cell lysis^13^, project outwards, so are in position to allow interactions with a lipid bilayer. By contrast, the loop is partially folded back in the structure of domain 4 alone (Fig. 1B), and fully bent back against the body of domain 4 in the previously determined structure of perfringolysin O^12^. Together the structures are consistent with previous suggestion that the loop springs outwards and downwards when the toxin binds to the membrane of a mammalian cell (Fig. 1B)^12^.

**Figure 2:**
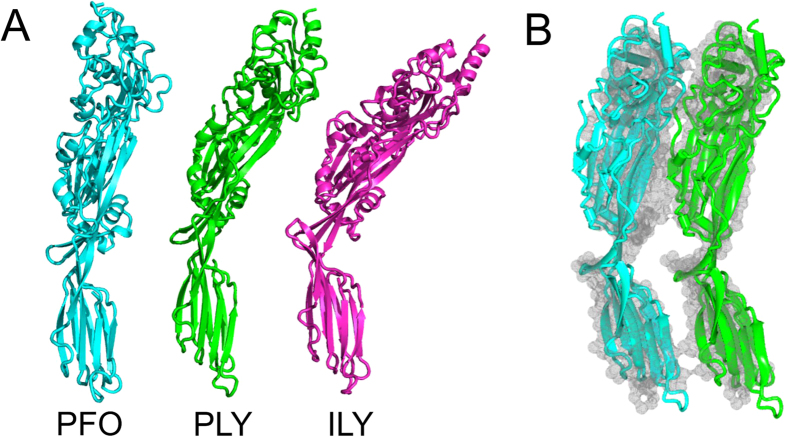
Packing of monomers in the pneumolysin crystal reveals the packing of the pre-pore complex. (**A**) Crystal structures of pneumolysin (PLY), intermedilysin (ILY) and perfringolysin (PFO) reveal differences in the angle between domains 1–3 and domain 4. (**B**) overlay of two fitted models into the prepore cryoEM density (*grey surface*; PDB: 2BK2^7^) with adjacent monomers from the crystal structure (*cyan* and *green*). The rms deviation was 4.90 Å (over 849 Cα atoms).

In the structure of full-length pneumolysin, domain 4 is offset by ~30° relative to the plane of the long axis of domains 1–3, such that the toxin resembles a bend rod from the side (Fig. 2A). The relative position of domain 4 differs in the available structures of CDCs. For example, it is displaced by ∼15 Å and twisted by ~25° in the crystal structure of perfrigolysin O (PDB: 1PFO^12^;), whereas it is offset even further (by ~40°) in intermedilysin (18; Fig. 2A). These differences suggest flexibility at the domain 2-domain 4 junction that would allow the toxin to dock onto the membrane surface and pack to form the pre-pore complex.

### The monomer/monomer interface

**Figure 3:**
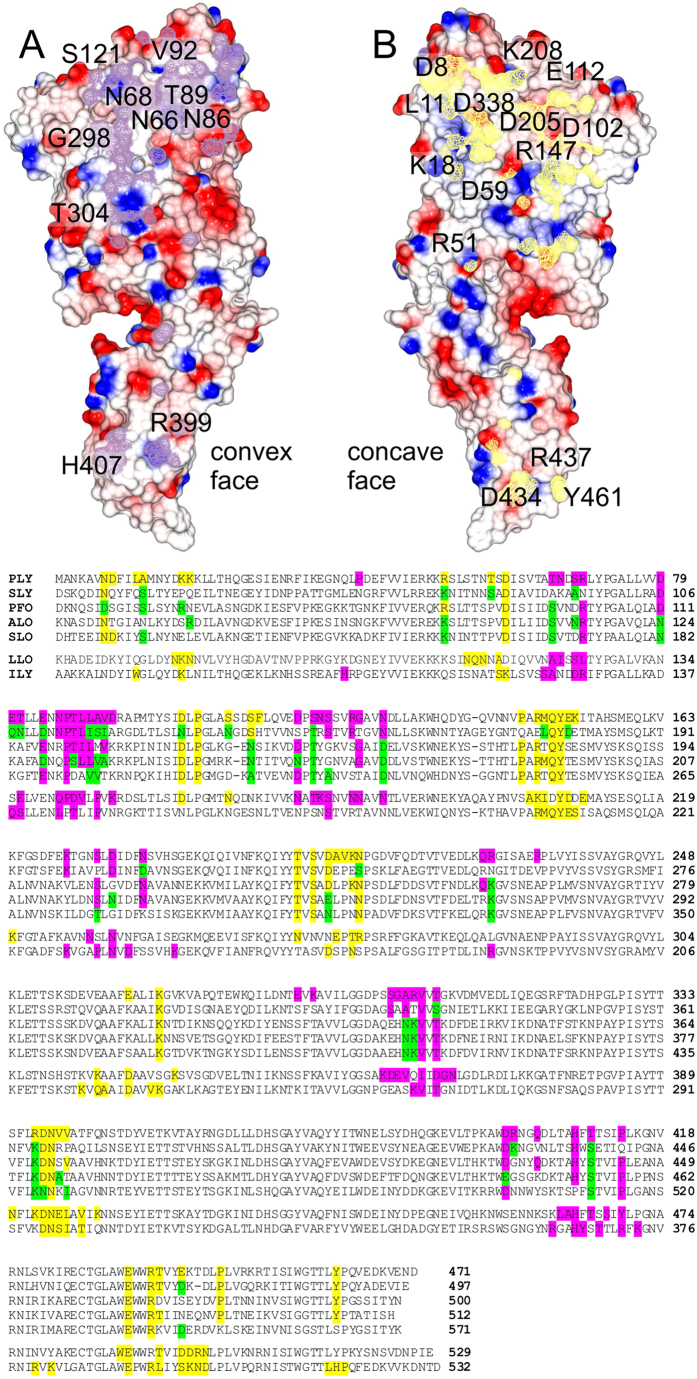
The conserved interface between adjacent pneumolysin monomers. (**A**) The convex and (B) concave interacting surfaces showing the electrostatic potential. Regions buried by the interaction are shaded in yellow and purple and key residues are labelled. (Lower panel) Alignment of the sequence of pneumolysin with other CDCs. Residues forming the interfaces in crystal structures of pneumolysin (PLY), intermedilysin (ILY) and lysteriolysin (LLO) are shaded as in *part* (A and B) above. Residues that are conserved in perfringolysin O (PFO), anthrolysin O (ALO) and suilysin (SLO) are shaded accordingly. Similar residues are in *green*.

A striking feature of pneumolysin crystals, is that monomers pack side-by-side in the crystal lattice, resembling the molecular packing of the pre-pore complex. Superimposition of two pneumolysin molecules from the crystal structure with adjacent monomers from the cryo-EM structure (PDB: 2BK2^7^) gives a RMS deviation of 4.90 Å (over 849 Cα atoms; Fig. 2B). Most notably, domain 4 is in the correct position for membrane binding relative to the rest of the toxin (Fig. 2B). The interface between pneumolysin molecules is created by the concave face of one monomer packing against the convex face of its neighbour. Although it encompasses almost the entire long axis of the toxin, only a relatively small subset of residues interact (Fig. 3A). Notably these could not be predicted from the cryoEM structures because the resolution is too low to see the side chains. Intermolecular contacts are made by all four domains, but mostly involve domains 1 and 4. There is some charge complementarity between the convex and concave faces, with the former being more negatively charged and the latter more positive (Fig. 3A). The interface includes 44 residues from the concave face and 41 residues in the convex face, with a total buried surface area of 939.6 Å^2^ and 962.8 Å^2^, respectively. Although most of the residues at the interface are polar, there are relatively few polar intermolecular interactions: salt bridges between Asp8 and Arg226 and Glu434 and Arg399 and hydrogen bonds between the side-chain of Asn339 and the carbonyl group of Pro87, and between the hydroxyl group of Tyr461 and the amine and carbonyl groups of His407. Thus, the major contribution towards oligomerization of monomers on the membrane is likely to be surface complementarity, leading to exclusion of water. Notably, of the 85 residues that are buried, 41 are identical and 63 are conserved in at least three of the other CDCs for which structures are available, suggesting that the interface is likely to be relatively well conserved in other CDCs (Fig. 3B). This conclusion is supported by comparison of the pneumolysin structure with the crystal structures of intermedilysin^19^ and listeriolysin O^20^, which also pack side-by-side in the crystal. Remarkably, contacts in the three structures involve equivalent regions of the CDC polypeptides, and many of the interacting residues are conserved (Fig. 3B).

### Mutation of residues at the monomer/monomer interface leads to loss of cytolytic activity

**Figure 4:**
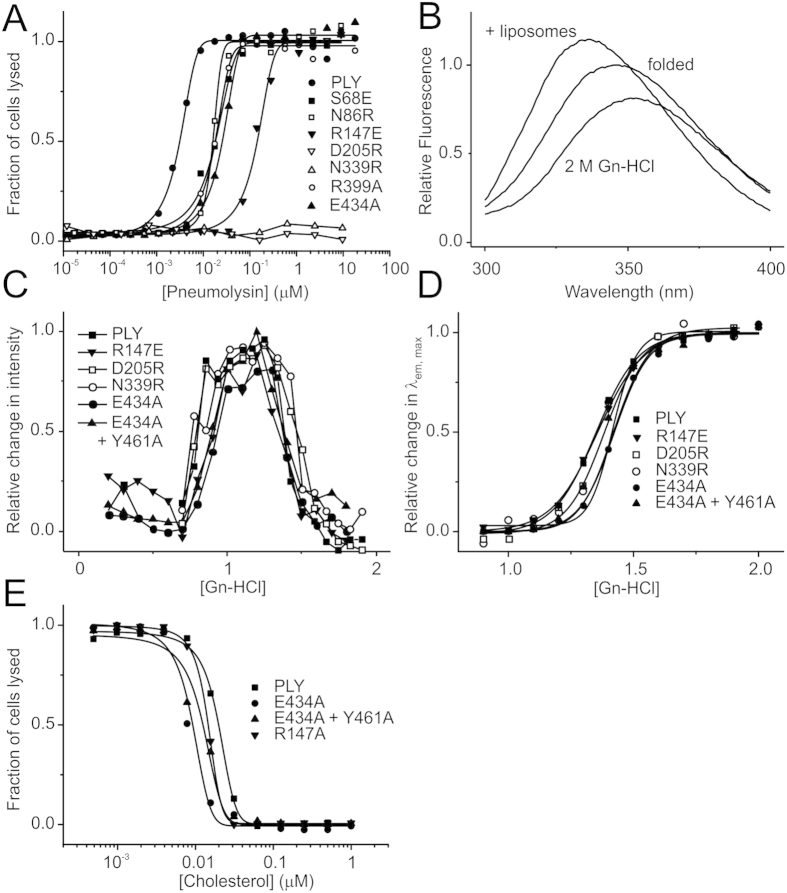
Hemolytic activities and biophysical properties of mutant pneumolysins. (**A**) hemolysis of sheep erythrocytes by selected pneumolysin mutants. (**B**) Fluorescence spectra of folded and unfolded pneumolysin (in 2M guanidine-HCl) and pneumolysin with liposomes. The fluorescence spectra of all mutants were comparable to that of the wild-type and exhibited similar changes with respect to the fluorescence intensity and λ_em, max_. (**C,D**), denaturation of wild-type and mutant pneumolysins measured by changes in fluorescence intensity (**C**) and λ_em, max_ (**D**). The unfolding transition measured by the change in λ_em, max_ corresponded approximately to the decrease in fluorescence observed in (**C**). (**E**) cholesterol-mediated inhibition of haemolysis by wild-type and mutant pneumolysins. Data are average of six measurements.

To test whether the intermolecular contacts observed in the crystal are important for the activity of the toxin, mutations were introduced to residues and the resulting cytolytic activity was measured using a simple haemolytic assay. In total, 18 single mutations were made to surface-exposed residues. Mutations Arg399Ala and Glu434Ala were designed to disrupt the salt bridge between adjacent membrane-binding domains and Tyr461Phe and Tyr461Ala to prevent the hydrogen bond between Tyr461 and the main chain of His407. Additional, mutations were introduced to disrupt the hydrophobic packing by removing side chains that pack against the adjacent monomer or by introducing large charged groups (Glu or Arg) at the interface. Leu11Arg, Ser68Glu, Asn86Arg, Thr88Glu, Arg147Ala, Asp205Arg Arg226Ala Thr304Arg, Asp338Ala, Ser68Glu, Asn86Arg, Arg147Glu, Asp338Arg were designed to disrupt the interface at the top of toxin (domains 1–3) and Arg437Ala to disrupt the domain 4/domain 4 interface. Although mostly polar in nature, these residues do not form intermolecular polar contacts, but rather exclude water at the interface Three double mutants were also created with mutations in domain 4: Arg339Ala + Glu434Ala; Glu434Ala + Tyr461Ala; and Glu434Ala + Arg437Ala. Importantly, with the exception of Arg147, which lies on the domains 1/3 boundary and Thr304 within domain 3, mutagenesis was restricted to residues in domains 1 and 4 that undergo relatively little positional changes upon pore formation. Regions undergoing large conformational changes, including residues 155–186 and 253–285 that form the membrane-spanning β-hairpins and domain 2, which rotates to allow the hairpins to penetrate the membrane, were excluded to ensure that the mutations did not prevent pore formation itself. All mutants were produced and purified as for wild-type pneumolysin. As with wild-type protein they eluted from a gel-filtration column as single peaks, corresponding to monomers, and circular dichroism and fluorescence spectroscopy (Fig. 4B) confirmed that they were folded.

**Figure 5:**
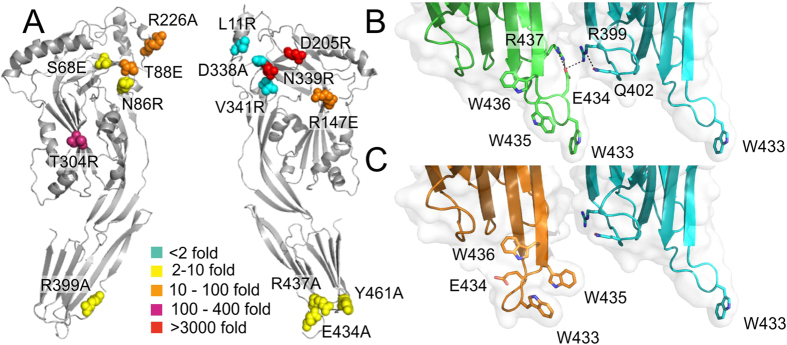
Contributions of residues towards haemolytic activity revealed by site directed mutagenesis. (**A**) Convex (*L*) and concave faces (*R*) of pneumolysin showing the positions of the mutations and the relative changes in hemolytic activity. (**B**) Packing between adjacent membrane-binding domains in the putative membrane binding conformation of pneumolysin (full-length pneumolysin structure). Glu434 forms a salt-bridge with Arg399 of the adjacent molecule and also orientates Arg437 to forms part of the monomer-monomer interface. (**C**) Substitution with the domain 4 structure (*orange*) reveals that the polar network is lost when the tryptophan-rich loop is fully or partially retracted as is predicted to occur in solution. In this way, membrane binding would promote oligomerization of pneumolysin monomers on the cell surface.

Almost all the mutations reduced the ability of pneumolysin to lyse sheep red blood cells, but by different degrees (Figs 4A and 5A and Table 2). The most striking effects were observed for the single mutations Asn339Arg and Asp205Arg, which failed to lyse sheep erythrocytes at the highest concentrations tested (10 μM) reflecting >3000-fold-fold decrease of hemolytic activity (Table 2). Both residues are located towards in the middle of the binding interface within domain 1 (Fig. 5A). Other mutations in domain 1 also lead to large decreases in activity, including Thr88Glu, Arg147E and Arg226Ala and to a lesser extent Ser68Glu and Asn86Arg. The mutation Thr304Arg within domain 3, also resulted in a large decrease in activity (300-fold). In the structure, the threonine side chain forms part of a β strand that packs against the hydrocarbon portion of the side chain of Lys268. This strand may displaced upon oligomerisation on the membrane to allow the monomers to pack together to form the ring. Only three mutations had relatively little effect on haemolysis (≤2-fold): Leu11Arg, Asp338Ala and V341Arg. Interestingly, all three residues are located in the posterior region of domain 1, which forms the outer part of the pre-pore ring, towards the perimeter of the binding interface. The clustering of these residues suggests that this region of the toxin may be less important for oligomerization, perhaps reflect some flexibility.

Mutations within domain 4 also reduced the haemolytic activity of pneumolysin. Replacement of Tyr461 with an alanine residue lead to a five-fold decrease in activity, whereas the Tyr461Phe mutation had little effect, suggesting that exclusion of water at the monomer interface is more important than the hydrogen bonds to His407. Disruption of the small polar network involving Glu434 and Arg437 of one monomer and Gln402 and R399 of the adjacent monomer (by mutations Arg437Ala, Glu434Ala or Arg399Ala) reduced the hemolytic activity by 6 to 10-fold. In the crystal structure, Glu434 not only forms a salt bridge with Arg399 but also interacts with Arg437 that in turn forms part of the binding interface by packing against the adjacent monomer. Interestingly, these interactions are only possible when the tryptophan-rich loop is flipped outwards in the putative membrane-binding conformation. When the loop is folded back, as in the domain 4 structure or in the structure of perfringolysin O (Fig. 1B), Glu434 no longer contacts Arg399 or Arg437 (Fig. 5B). This mechanism would help to stabilise oligomerization on the membrane surface but not in solution. As expected, the Glu434Ala + Arg437Ala double mutant is even more disrupted than the single mutations (30-fold loss in activity). The other double mutations are similarly affected with 20- to 30-fold decreases in activity (Table 1).

To confirm that the mutations did not impair hemolytic activity through destabilisation or changes to membrane binding, we compared the stabilities and membrane binding properties of five of the mutants with the largest decreases in hemolytic activity: Asn339Arg, Asp205Arg, Glu434Ala, Arg147Glu and the double mutant Glu434Ala + Tyr461Ala. Stabilities were examined by guanidine-HCl denaturation and measured by fluorescence. Pneumolysin has a complex denaturation profile, probably reflecting its multidomain structure in which the fluorescence intensity increased over the range 0.75–1.25 M guanidine-HCl and then decreased from 1.25–1.75 M. Further increases in the concentration of denaturant resulted in no further changes, suggesting that pneumolysin was already fully unfolded (Fig. 4C). The decrease in fluorescence intensity was accompanied by a shift in the λ_em, max_ from 347 nm to 354 nm, consistent with buried tryptophan residues becoming exposed to the polar solvent. Plotting the λ_em, max_ against the concentration of guanidine-HCl gave a simple two-state transition in which the midpoint was ~1.4 M guanidine-HCl (Fig. 4D). The five mutants all had comparable denaturation curves both with respect to fluorescence intensity (Fig. 4C) and λ_em, max_ (Fig. 4D), indicating that they are not destabilised by the mutations.

To investigate membrane binding, we added pneumolysin to cholesterol-containing liposomes using fluorescence to detect any changes that occur. Liposome binding by the wild-type toxin lead to a small increase (~15%) in the fluorescence intensity that was accompanied by an decrease in the λ_em, max_ from 347 to 342 nm (Fig. 4B), presumably reflecting the tryptophan-rich loop becoming buried into the hydrophobic lipid bilayer. All the mutants with reduced hemolytic activities were tested in this way and showed comparable fluorescence changes as the wild-type toxin, both with respect to fluorescence intensity and λ_em, max_. Although, it was not possible to measure the binding affinities in this assay, these data indicate that the mutant toxins bind to cholesterol-containing lipid bilayers in a similar way to the wild-type toxin. To further examine lipid binding, a hemolytic inhibition assay was used for the 3 mutants that showed some residual haemolytic activity. In this assay, pneumolysin was incubated with increasing concentrations of cholesterol prior to the addition of sheep erythrocytes. Cholesterol inhibited hemolysis by wild-type pneumolysin with an IC_50_ of 20 nM. IC_50_ values of 9, 14 and 15 nM were measured for the Glu434Ala, Arg147Glu and Glu434Ala + Tyr461Ala mutants, respectively, indicating that they bind cholesterol with comparable affinities to the wild-type toxin. Taken together, these data show that the loss in hemolytic activity is not caused by changes in stability or cholesterol binding. Thus, the mutations probably disrupt intermolecular packing of pneumolysin monomers on the membrane surface prior to pore formation.

## Discussion

It has been shown that pneumolysin oligomerises spontaneously in solution but only at high protein concentrations, revealing weak interactions between monomers, even in the absence of a cholesterol-containing lipid bilayer^21^,^22^. We propose that these interactions have been captured in the crystals of pneumolysin and this conclusion is strengthened by the structures of intermediolysin and listeriolysin O, which pack together in a similar way. The structure reveals shape and charge complementarity between adjacent pneumolysin monomers in which a concave, more-negatively charged surface packs against a convex more positive face. The buried surface is modest compared to stable protein-protein complexes^23^, as would be expected for a protein that is predominantly monomeric at physiological concentrations. Upon binding to a lipid bilayer however, diffusion in only two possible planes would favour oligomerisation to form the pre-pore. Self-association is likely to be further favoured by additional interactions that only occur once pneumolysin monomers have bound to the membrane surface. Thus, the small polar network involving Glu434 and Arg437 of one monomer and Gln402 and Arg399 of its neighbour is only possible when the tryptophan-rich flips outwards in the putative membrane-binding conformation (Fig. 5B). In solution, Glu434 does not contact Arg437, so is unable to contribute towards monomer-monomer packing.

Monomers are parallel in the crystal structure but must be slightly offset on the cell membrane to create the ring-shaped pre-pore and pore oligomers. Furthermore, pores vary in size, typically with between 30–50 subunits, so there must be some tolerance in the intermolecular packing (ranging from an offset of 12° in a 30mer to 7.2° in a 50mer). Notably, most of the packing interactions in the crystal are within the central portion of the binding interface (rather than towards the edges, which would tend to lock the interaction) and are therefore likely to allow some leeway in the angle between monomers.

Most of the contacts between pneumolysin monomers are between residues in domains 1 and 4, at the top and bottom of the toxin, respectively. These domains are believed to change conformation the least during pore formation, with domain 4 serving as a fixed membrane anchor throughout and domain 1 acting as a bridge between domain 2, which rotates as the pre-pore collapses down to form the pore, and domain 3, which rearranges to form the β-hairpins that penetrate the membrane^7^. It is likely therefore that at least some of the intermolecular contacts involving domains 1 and 4 are retained within the pore state, thereby stabilising the oligomers during and after the major conformational change.

Recently it has emerged that pneumolysin and several other CDC are lectins that probably have dual receptors on host cells involving a carbohydrate ligand as well as cholesterol^24^. Pneumolysin binds to sialyl-Lewis X on red blood cells. Significantly, the putative carbohydrate-binding residues that were identified in this study are not involved in or buried by the intermolecular interactions identified in our work, allowing simultaneous receptor binding and pre-pore formation on the membrane surface.

Pneumolysin is a target for inhibitors for the treatment of pneumococcal disease because of its importance for pneumococcal pathogenicity and its conservation in the different *S. pneumoniae* serotypes. Disruption of pneumolysin oligomerisation is an attractive potential strategy for the future design of such inhibitors and the structure described here forms the basis for rational approaches.

## Methods

### Production and purification of wild-type and mutant pneumolysin and domain 4

The gene encoding pneumolysin from serotype D39 was amplified by PCR and cloned into the polylinker of a modified pET-based expression vector. Protein was synthesised with an N-terminal His_6_ tag. Freshly transformed *E. coli* BL21 (DE3) were grown to an OD_600_ of 0.6–0.8, induced with 0.1 mM IPTG and incubated at 18 °C, with shaking at 220 rpm overnight. Cells were harvested by centrifugation at 3,500 g for 15 minutes at 4 °C and resuspended in lysis buffer (20 mM Tris-HCl, 150 mM NaCl, 20 mM imidazole and 1% v/v Tween 20 at pH 7.5) with a protease inhibitor tablet (Roche). Cells were lysed by sonication and cell debris was removed by centrifugation. The supernatant was then loaded onto an nickel-Sepharose column (5 ml; GE healthcare), pre-equilibrated with lysis buffer. After loading, the column was washed with 50 ml lysis buffer without Tween 20 and pneumolysin was eluted using a linear gradient of imidazole between 0 and 1 M. Pneumolysin was further purified by gel filtration using a Superdex 200 16/60 column in 20 mM Tris-HCl at pH 7.5. Domain 4 was expressed as a maltose-binding protein fusion and purified by affinity chromatography on a amylose-Sepharose column (1 ml; GE healthcare). Domain 4 was released by overnight digestion with TEV protease and separated from maltose-binding protein by gel-filtration on a Superdex 200 16/60 column. Mutations were introduced into pneumolysin by PCR and proteins were expressed and purified as for the wild-type toxin.

### Crystallization and data collection

Pneumolysin was crystallised using the sitting-drop vapor diffusion method by mixing equal volumes of pneumolysin (10 mg/mL) and 100 mM Tris-Bicine pH 8.5, containing 30 mM CaCl_2_, 30 mM MgCl_2_, 20% v/v glycerol, 14% w/v PEG 4K, 6 mM phosphocholine chloride at 4 °C. Domain 4 was crystallised in 30% w/v PEG 2000 MME containing 100 mM potassium thiocyanate at 20 °C. All crystals were stored in liquid nitrogen and were maintained at 100 K during data collection. Diffraction data were collected at Diamond Light Source and were processed with Mosflm^25^. Phases for the full length protein were determined by molecular replacement with Phaser^26^ using the structure of perfringolysin as a search model (PDB: 1M3I). Phases for the domain 4 structure were determined using domain 4 of the pneumolysin structure as a search model. Models were optimized by using cycles of manual refinement with Coot^27^ and refinement in Refmac5, part of the CCP4 software suite^28^, and in Phenix^29^. Both wild-type pneumolysin and pneumolysin containing the double substitution Asp385Asn and Cys432Ala were crystallised, but the mutant consistently gave the best quality crystals.

### Hemolytic assays

Sheep red blood cells (1% v/v; Thermo Scientific) were washed by pelleting 10 ml defibrinated sheep blood at 1734 g, at 4 °C and resuspending the cell pellet in chilled phosphate-buffered saline. Two-fold serial dilutions of pneumolysin in phosphate-buffered saline was aliquoted into a round-bottomed 96-well microtitre plate, and mixed with an equal volume of 1% v/v sheep blood. The plates were incubated for 30 minutes at room temperature, and intact red blood cells and cellular debris were pelleted by centrifugation at 1734 g, at 4 °C. The supernatant was transferred to corresponding wells in a flat-bottomed 96-well microtitre plate and the absorbance of each well was measured at 410 nm. Inhibition assays were carried out in a similar way except that dilutions of cholesterol were added to a fixed amount of pneumolysin (0.626 μM). This concentration of pneumolysin was sufficient to lyse the erythrocytes even for the mutants with reduced haemolytic activity.

### Fluorescence

Fluorescence data were collected in a Fluoromax-4 (Horiba Jobin Yvon) instrument at a final protein concentration of 1 μM pneumolysin, using a low-volume quartz fluorescence cuvette (300 μl). Proteins were excited at 280 nm with a 2.5 nm slit width and fluorescence was detected between 300 and 400 nm at 1 nm intervals with a 2.5 nm slit width. All spectra were collected at 20 °C. Samples were allowed to equilibrate for 150 seconds before spectra were collected and buffer blanks were subtracted from all datasets.

### Liposomes

Liposomes were generated by addition of L-α-phosphatidylcholine (130 μmoles; 100 mg) to cholesterol (130 μmoles; 50 mg) and dihexadecyl phosphate (13 μmoles; 7.1 mg) to 5 ml of a 1:1 methanol/chloroform mix in a glass volumetric flask (5 ml). The mixture was solubilized by agitation and the liquid removed under a dry stream of nitrogen on ice. The nitrogen was stopped once all visible liquid had evaporated and the lipids were a translucent yellow gel sticking to the sides of the flask. The lipids were suspended in 5 ml of PBS by vortexing and then sonicating at room temperature, for 90 seconds.

## Additional Information

**How to cite this article**: Marshall, J. E. *et al.* The Crystal Structure of Pneumolysin at 2.0 Å Resolution Reveals the Molecular Packing of the Pre-pore Complex. *Sci. Rep.* **5**, 13293; doi: 10.1038/srep13293 (2015).

## Figures and Tables

**Table 1:** Data collection and refinement statistics.

	Pneumolysin	Pneumolysin domain 4
Data collection		
PDB ID	Will be included in final version	Will be included in final version
Beam Line	Diamond I04-1	Diamond I03
Space group	*P* 2_1_ 2_1_ 2_1_	*I*121
a,b,c, Å	27.1, 128.1, 172.1	48.7, 47.7, 97.5
α, β,γ , °	90, 90, 90	90, 101.4, 90
Resolution, Å	43.07 – 1.94 (1.99 – 1.94)	46.53 – 2.05 (2.12 – 2.05)
*R*_*sym*_	0.044 (0.461)	0.091 (0.451)
*I/σ(I)*	39.7 (5.84)	6.8 (2.0)
Completeness	99.4 (99.9)	99.4 (97.8)
Redundancy	6.2 (6.3)	2.9 (2.4)
Refinement		
Resolution	43.07 – 1.98 (2.05 – 1.98)	46.53 – 2.05 (2.12 – 2.05)
No. reflections	42799 (4227)	40468 (2447)
*R*_*work*_*/R*_*free*_	0.246/0.269	0.218/0.271
No of atoms	3856	2021
Protein	3661	1870
water	195	151
B-factors, Å^2^	67.2	35.0
Protein	68.1	35.1
water	51.4	33.6
Rms deviations		
Bond lengths, Å	0.004	0.004
Bond angles, °	0.81	0.83

The highest resolution shell is shown in parentheses.

**Table 2:** Hemolytic activities of pneumolysin mutants.

Protein	EC_50_ (nM)	Relative Hemolytic Activity
Wild type PLY	2.6 ± 0.5	1.0
Leu11Arg	4.6 ± 1.6	0.69 ± 0.35
Ser68Glu	16.2 ± 1.6	0.16 ± 0.03
Asn86Arg	14.7 ± 1.5	0.18 ± 0.03
Thr88Glu	76 ± 20	0.039 ± 0.017
Arg147Ala	11 ± 1	0.24 ± 0.03
Arg147Glu	115 ± 2	0.023 ± 0.002
Asp205Arg	>10000	<0.0003
Arg226Ala	48 ± 16	0.064 ± 0.031
Thr304Arg	900 ± 200	0.0031 ± 0.0013
Asp338Ala	2.9 ± 1.1	1.1 ± 0.3
Asp338Arg	1.4 ± 0.4	0.53 ± 0.11
Asn339Arg	>10000	<0.0003
Val341Arg	2.5 ± 1.0	1.3 ± 0.8
Arg399Ala	15 ± 0.91	0.17 ± 0.02
Glu434Ala	22 ± 1	0.12 ± 0.02
Arg437Ala	21 ± 1	0.13 ± 0.01
Tyr461Ala	20 ± 1	0.13 ± 0.02
Tyr461Phe	1.3 ± 0.1	2.0 ± 0.3
Arg339Ala + Glu434Ala	48 ± 1	0.051 ± 0.003
Glu434Ala + Tyr461Ala	86 ± 1	0.030 ± 0.003
Glu434A + Arg437Ala	84 ± 4	0.030 ± 0.004

Values are mean ± error from two experiments.
